# Symptomatic MERS-CoV infection reduces the risk of future COVID-19 disease; a retrospective cohort study

**DOI:** 10.1186/s12879-023-08763-2

**Published:** 2023-11-03

**Authors:** Aiman El-Saed, Fatmah Othman, Henry Baffoe-Bonnie, Rawabi Almulhem, Muayed Matalqah, Latifah Alshammari, Majid M. Alshamrani

**Affiliations:** 1 Infection Prevention and Control Department, King Abdulaziz Medical City, Riyadh, Saudi Arabia; 2 King Saud bin Abdulaziz University for Health Sciences, Riyadh, Saudi Arabia; 3 Present Address: King Abdullah International Medical Research Center, Riyadh, Saudi Arabia; 4 Epidemiology and Biostatistics Department, College of Public Health and Health Informatics, King Saud bin Abdul Aziz University for Health Science, Riyadh, Saudi Arabia; 5 Infection Prevention and Control Department, King Abdullah Specialist Children Hospital, Riyadh, Saudi Arabia

**Keywords:** MERS-CoV, COVID-19, Cross-immunity, Cross-protection, Saudi Arabia

## Abstract

**Background:**

The general human immune responses similarity against different coronaviruses may reflect some degree of cross-immunity, whereby exposure to one coronavirus may confer partial immunity to another. The aim was to determine whether previous MERS-CoV infection was associated with a lower risk of subsequent COVID-19 disease and its related outcomes.

**Methods:**

We conducted a retrospective cohort study among all patients screened for MERS-CoV at a tertiary care hospital in Saudi Arabia between 2012 and early 2020. Both MERS-CoV positive and negative patients were followed up from early 2020 to September 2021 for developing COVID-19 infection confirmed by RT-PCR testing.

**Results:**

A total of 397 participants followed for an average 15 months during COVID-19 pandemic (4.9 years from MERS-CoV infection). Of the 397 participants, 93 (23.4%) were positive for MERS-CoV at baseline; 61 (65.6%) of the positive cases were symptomatic. Out of 397, 48 (12.1%) participants developed COVID-19 by the end of the follow-up period. Cox regression analysis adjusted for age, gender, and major comorbidity showed a marginally significant lower risk of COVID-19 disease (hazard ratio = 0.533, p = 0.085) and hospital admission (hazard ratio = 0.411, p = 0.061) in patients with positive MERS-CoV. Additionally, the risk of COVID-19 disease was further reduced and became significant in patients with symptomatic MERS-CoV infection (hazard ratio = 0.324, p = 0.034) and hospital admission (hazard ratio = 0.317, p = 0.042).

**Conclusions:**

The current findings may indicate a partial cross-immunity, where patients with symptomatic MERS-CoV have a lower risk of future COVID-19 infection and related hospitalization. The present results may need further examination nationally using immunity markers.

## Introduction

Since the emergence of COVID-19 in December 2019, a significant global public health pandemic emergency has imposed many challenges on healthcare sectors worldwide [1]. This largest pandemic was caused by severe acute respiratory syndrome (SARS-CoV-2), a virus belonging to a large family of single-stranded RNA coronaviruses that mainly cause respiratory tract infections [2, 3]. The clinical importance and the epidemic possibility of coronaviruses started to be recognized in 2002 when the severe acute respiratory syndrome coronavirus (SARS-CoV-1) epidemic occurred, with 66% of the cases detected in China [4]. Subsequently, this was followed by the middle east respiratory syndrome coronavirus MERS-CoV infection in 2012, with more than 80% of the cases detected in Saudi Arabia [5, 6].

Although suggested, it is still unclear whether coronaviruses have cross-reactive immunity between different types [7–9]. A growing body of evidence indicates that coronavirus infection triggers both humoral and cellular immunities essential to eliminate the viral infection [10, 11]. Sero-prevalence studies showed that the detection of antibodies against coronaviruses starts early during the disease, after 11 days in SARS-CoV-2, 16 days in MERS-CoV, and 12 days in SARS-CoV-1 [9, 10, 12]. SARS-CoV-2 specific antibody levels declined to undetectable levels after two to three months in 40% of asymptomatic and 13% of symptomatic individuals [13]. This decline of antibody levels is much quicker than in MERS-CoV infection, where the specific antibody responses can persist for 2–6 years in patients who survived a severe form of the disease and to a shorter duration in patients with a subclinical or mild form of MERS-CoV disease [14–16]. However, it is strongly believed that cellular immunity is essential for long-term immunity in both SARS-CoV-2 [17] and MERS-CoV [18].

The general similarity of the immune responses against different coronaviruses may suggest the presence of some degree of cross-immunity, with exposure to one virus may confer partial immunity to another [8, 11]. Even at the level of testing, there has been some degree of cross-reactivity between different coronaviruses [8, 9]. This study aimed to determine whether previous MERS-CoV infection was associated with a lower risk of subsequent COVID-19 disease and COVID-19-related outcomes, including disease severity and hospitalization.

## Methods

### Study design

A retrospective cohort study was carried out among all patients screened for MERS-CoV at King Abdulaziz Medical City in Riyadh (KAMC-R) in Saudi Arabia between September 2012 and March 2020. Both MERS-CoV positive and negative cohorts were followed up through September 2021 for the development of COVID-19 infection. The study obtained all required ethical approvals.

### Setting

The study was conducted at KAMC-R, Saudi Arabia. KAMC-R is an approximately 1488-bed tertiary care facility composed of two hospitals. KAMC-R provides healthcare services for almost 1.15 million eligible Saudi National Guard soldiers, employees, and their families. The Medical City is Joint Commission International (JCI) accredited facility.

### Case finding

Per the Saudi Ministry of Health (MOH) regulations, the basic information of all PCR-confirmed MERS-CoV patients is reported electronically through the Saudi Health Electronic Surveillance Network (HESN). The HESN reporting system is an integrated national electronic surveillance system governed by MOH in Saudi Arabia. HESN has several domains to uniformly monitor infectious diseases, disease epidemics, immunization, and Hospital Acquired Infections across Saudi Arabia [19]. Eligibility criteria were verified by utilizing both HESN basic information and local infection control data at KAMC-R.

### Study population

All patients screened for MERS-CoV between September 2012 and March 2020, irrespective of their test results, age, gender, nationality, and employment status (healthcare versus non-healthcare provider), were identified. Patients who died after MERS-CoV testing and before March 2020 and those who had less than three months between MERS-CoV testing and COVID-19 assessment were excluded. Additionally, those who were missing testing results of either MERS-CoV or COVID-19 were excluded from this study.

### Sample size and sampling

According to WHO reports, 1333 patients with MERS-CoV in Saudi Arabia survived the disease by May 2020 [6], and less than 10% were diagnosed in KAMC-R. Given the presence of a small population of patients with positive MERS-CoV at KAMC-R (N = 100), it was estimated that 90 patients would be required to detect 10% (± 2.0%) of COVID-19 infection. Additionally, 270 individuals with negative MERS-CoV (in a ratio of 3:1) were recruited as a comparison group, to adjust for the small number of patients with positive MERS-CoV. Therefore, the total sample size was 360 patients (90 positive and 270 negative MERS-CoV). The negative cohort was group-matched with the positive cohort as regards the year of testing, age (within five years), and gender. Given the limited number of survived MERS-CoV patients who have COVID-19 status in our center, all eligible patients were included in the study and no special sampling was done.

### Data collection tool

Detailed information on both cohorts was collected using a standardized data collection form. These included demographic characteristics, working status (health care workers versus patients), and disease severity during MERS-CoV assessment. Additionally, medical comorbidities and COVID-19 status and severity at the end of the follow-up duration.

### Exposure and outcome definitions

Laboratory-confirmed MERS-CoV patients (exposure groups) and Laboratory-confirmed COVID-19 patients (outcome groups) were determined using reverse transcription-polymerase chain reaction (RT-PCR) testing. Other outcomes were investigated, including COVID-19 severity, complications, mortality, hospital/ICU admission, use and duration of Ventilation, and hospital/ICU length of stay. The severity of MERS-CoV disease was categorized as asymptomatic, mild/moderate (home isolation vs. hospital ward admission), and severe (required ICU admission). The severity of COVID-19 disease was categorized as asymptomatic, mild (symptomatic without evidence of pneumonia or hypoxia), moderate (clinical signs of pneumonia but no hypoxia), severe (severe pneumonia or hypoxia), and acute respiratory distress syndrome (ARDS).

### Statistical analysis

Continuous variables were expressed as mean and standard deviation (SD) or median with appropriate interquartile ranges (IQRs), whereas categorical variables were expressed as numbers and percentages. Additionally, the incidence of the study outcomes per 1000 patient years was calculated. The study outcomes were compared between patients with positive versus negative MERS-CoV and patients with symptomatic versus negative or asymptomatic MERS-CoV infection. Significant differences between groups were examined using Chi-square or Fisher’s exact tests as appropriate for categorical variables, t-test or Mann-Whitney test as appropriate for continuous variables, and Z-test for incidence. Crude and adjusted (multivariate) Cox regression models predicting the study outcomes at the end of the follow-up period by baseline MERS-CoV status were run to estimate the hazard ratio (HRs) and COVID-19-free survival. Multivariate models were adjusted for age at COVID, gender, and significant comorbidity (hypertension, diabetes, heart diseases, lung diseases, and renal disease/hemodialysis). P-values < 0.05 were considered significant. SPSS (Version 27.0. Armonk, NY: IBM Corp) was used for all statistical analyses.

## Results

**Fig. 1:**
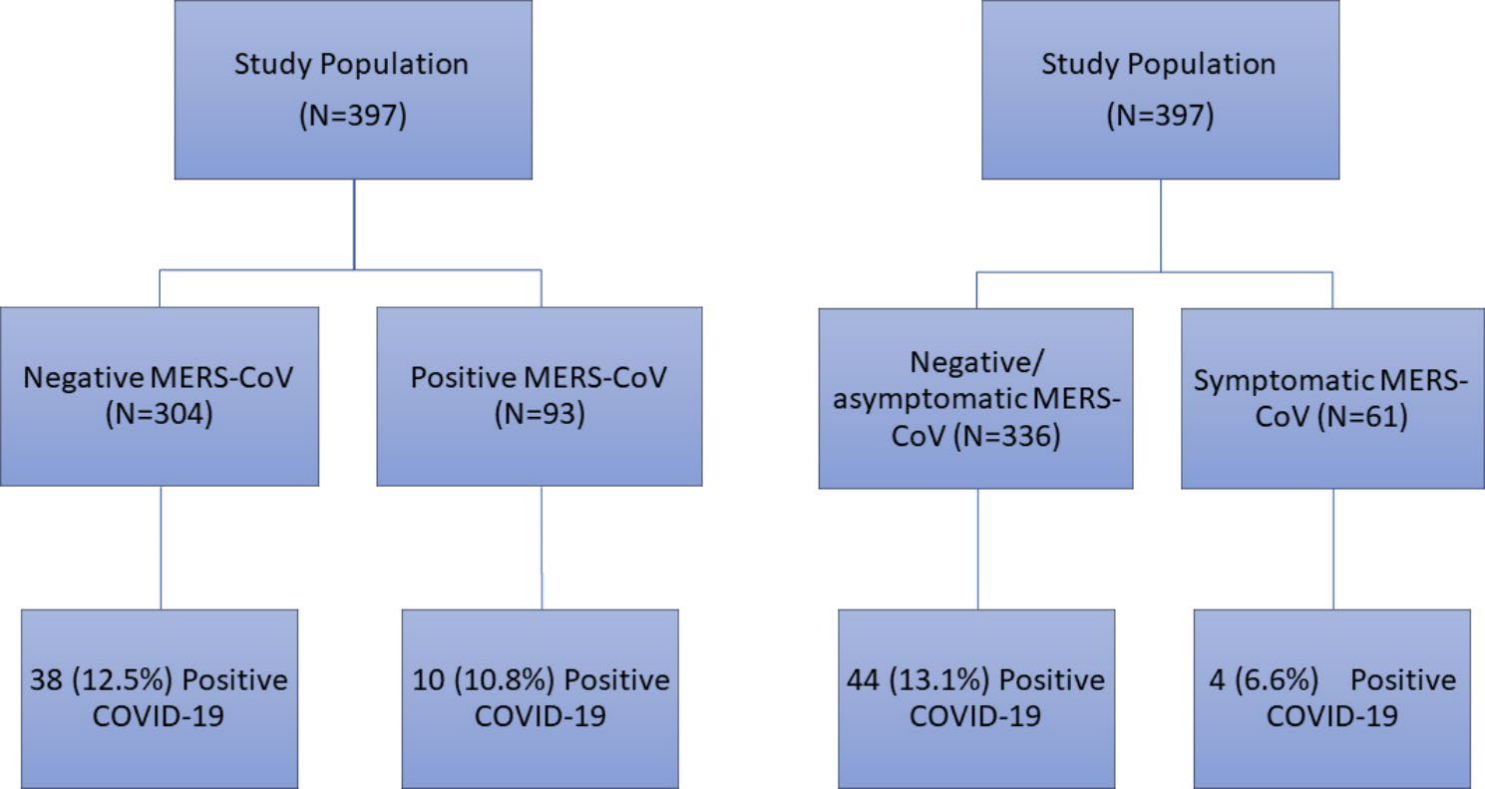
Diagram of developing COVID-19 infection by MERS-CoV status

Out of 418 patients, 21 (5.0%) patients were excluded for either short (< 3 months) follow-up duration (N = 9, 2.2%) or lack of information about MERS-CoV proof (N = 5, 1.2%) or COVID-19 proof (N = 7, 1.7%). A total of 397 were included in the current analysis, and they were followed for an average 15 months during COVID-19 pandemic, which was on average 4.9 years from the MERS-CoV infection. Out of the included patients, 93 (23.4%) had positive MERS-CoV PCR tests, 61 (15.4%) had symptomatic MERS-CoV infection, and 48 (12.1%) had positive COVID-19 PCR tests (Fig. 1).

### Description of the population at baseline

As shown in Table 1, the mean age was 46.4 ± 19.3 years. Approximately 54.8% were females, 62.8% were Saudi, and 40.6% were healthcare workers. The main presentations were respiratory symptoms (37.3%) and fever (23.9%), while almost half (51.6%) of patients were asymptomatic. Approximately 8.8% required ICU admission, and 2.8% required Mechanical Ventilation. MERS-CoV was significantly associated with non-Saudi patients, healthcare workers, having respiratory symptoms or fever, having severe disease, and requiring ICU admission or Mechanical Ventilation.

**Table 1:** Demographic and clinical characteristics of the study population at baseline by MERS-CoV status

	MERS-CoV status			P-value
	No (N = 304)	Yes (N = 93)	Total (N = 397)	
**Age at MERS-CoV testing**	46.6 ± 20.5	46.0 ± 15.0	46.4 ± 19.3	0.791
**Gender**				
Male	130 (43.9%)	46 (49.5%)	176 (45.2%)	0.349
Female	166 (56.1%)	47 (50.5%)	213 (54.8%)	
**Nationality**				
Non-Saudi	100 (33.4%)	46 (49.5%)	146 (37.2%)	0.005
Saudi	199 (66.6%)	47 (50.5%)	246 (62.8%)	
**Healthcare workers status**				
Patient	186 (62.0%)	47 (51.1%)	233 (59.4%)	0.062
Employee	114 (38.0%)	45 (48.9%)	159 (40.6%)	
**Symptoms**				
Fever	62 (20.4%)	33 (35.5%)	95 (23.9%)	0.003
Respiratory	100 (32.9%)	48 (51.6%)	148 (37.3%)	0.001
Gastrointestinal	17 (5.6%)	8 (8.6%)	25 (6.3%)	0.296
Constitutional	20 (6.6%)	7 (7.5%)	27 (6.8%)	0.751
**Severity of disease**				
Asymptomatic	173 (56.9%)	32 (34.4%)	205 (51.6%)	< 0.001
Mild/moderate (home isolation or wad stay)	125 (41.1%)	37 (39.8%)	162 (40.8%)	
Severe (required ICU stay)	6 (2.0%)	24 (25.8%)	30 (7.6%)	
**ICU outcomes**				
ICU admission	12 (3.9%)	23 (24.7%)	35 (8.8%)	< 0.001
ICU ventilation	3 (1.0%)	8 (8.6%)	11 (2.8%)	< 0.001
**Duration of stay** (median and IQR)				
ICU length of stay (days)	10 (5–22)	15 (6–34)	15 (6–22)	0.666
ICU ventilator days	1 (1–1)	13 (5–61)	11 (2–39)	0.143

### Description of patients with COVID-19

As shown in Table 2, the mean age was 51.2 ± 19.7 years. Approximately 54.2% were females, 70.8% were Saudi, and 31.3% were healthcare workers. The main presentations were respiratory symptoms (66.7%), and fever (33.3%), with the majority (77.1%) of patients having mild/moderate disease (no hypoxia). The main complications were an acute respiratory failure (8.3%) and septic shock (6.3%). The main comorbidity included hypertension (35.4%), diabetes (29.2%), heart diseases (14.6%), lung diseases (14.6%), and renal disease, including hemodialysis (14.6%). Approximately 27.1% required hospital admission, 8.3% needed ICU admission, and 4.2% required Mechanical Ventilation. Diagnosing COVID-19 was significantly associated with developing respiratory symptoms, fever, constitutional symptoms, severe disease, acute respiratory failure, or septic shock, and requiring hospital or ICU admission.

**Table 2:** Demographic and clinical characteristics of the study population at the end of follow up by COVID-19 status

	COVID-19 status			P-value
	No (N = 349)	Yes (N = 48)	Total (N = 397)	
**Age at COVID-19 testing**	51.0 ± 18.9	51.2 ± 19.7	51.0 ± 19.0	0.944
**Gender**				
Male	154 (45.2%)	22 (45.8%)	176 (45.2%)	0.930
Female	187 (54.8%)	26 (54.2%)	213 (54.8%)	
**Nationality**				
Non-Saudi	132 (38.4%)	14 (29.2%)	146 (37.2%)	0.217
Saudi	212 (61.6%)	34 (70.8%)	246 (62.8%)	
**Healthcare workers status**				
Patient	200 (58.1%)	33 (68.8%)	233 (59.4%)	0.161
Employee	144 (41.9%)	15 (31.3%)	159 (40.6%)	
**Symptoms**				
Fever	8 (2.3%)	16 (33.3%)	24 (6.0%)	< 0.001
Respiratory	22 (6.3%)	32 (66.7%)	54 (13.6%)	< 0.001
Gastrointestinal	9 (2.6%)	3 (6.3%)	12 (3.0%)	0.167
Constitutional	6 (1.7%)	5 (10.4%)	11 (2.8%)	0.006
**Severity of disease**				
Asymptomatic	310 (88.8%)	4 (8.3%)	314 (79.1%)	< 0.001
Mild/moderate	35 (10.0%)	37 (77.1%)	72 (18.1%)	
Severe/ARDS	4 (1.1%)	7 (14.6%)	11 (2.8%)	
**Complications**:				
Acute respiratory failure	3 (0.9%)	4 (8.3%)	7 (1.8%)	0.005
Acute kidney injury	4 (1.1%)	1 (2.1%)	5 (1.3%)	0.477
Cardiovascular complications	3 (0.9%)	1 (2.1%)	4 (1.0%)	0.404
Septic shock	0 (0.0%)	3 (6.3%)	3 (0.8%)	0.002
Venous thromboembolism	1 (0.3%)	0 (0.0%)	1 (0.3%)	> 0.99
Acute liver injury	1 (0.3%)	0 (0.0%)	1 (0.3%)	> 0.99
Disseminated intravascular coagulation	1 (0.3%)	0 (0.0%)	1 (0.3%)	> 0.99
Other complication	5 (1.4%)	2 (4.2%)	7 (1.8%)	0.203
**Comorbidity**				
Hypertension	109 (31.2%)	17 (35.4%)	126 (31.7%)	0.559
Diabetes	81 (23.2%)	14 (29.2%)	95 (23.9%)	0.364
Heart diseases	43 (12.3%)	7 (14.6%)	50 (12.6%)	0.658
Lung diseases	29 (8.3%)	7 (14.6%)	36 (9.1%)	0.177
Renal disease/hemodialysis	33 (9.5%)	7 (14.6%)	40 (10.1%)	0.303
Metabolic disease	19 (5.4%)	5 (10.4%)	24 (6.0%)	0.191
Neurological disease	16 (4.6%)	1 (2.1%)	17 (4.3%)	0.706
Cancer	15 (4.3%)	2 (4.2%)	17 (4.3%)	> 0.99
Other comorbidity (all)	55 (15.8%)	7 (14.6%)	62 (15.6%)	> 0.99
**Outcomes**				
Hospital admission	19 (5.4%)	13 (27.1%)	32 (8.1%)	< 0.001
ICU admission	5 (1.4%)	4 (8.3%)	9 (2.3%)	0.015
ICU ventilation	3 (0.9%)	2 (4.2%)	5 (1.3%)	0.113
**Duration** (median and IQR)				
Hospital length of stay (days)	8 (2–20)	4 (3–17)	6 (2–18)	0.679
ICU length of stay (days)	15 (8–18)	16 (8–16)	16 (9–17)	> 0.99
ICU ventilator days	11 (7-.)	11 (5-.)	11 (6–17)	0.564
Follow up years	5.0 (3.7–5.9)	4.2 (3.0-5.1)	4.9 (3.5–5.8)	0.002

### Crude outcomes

**Fig. 2:**
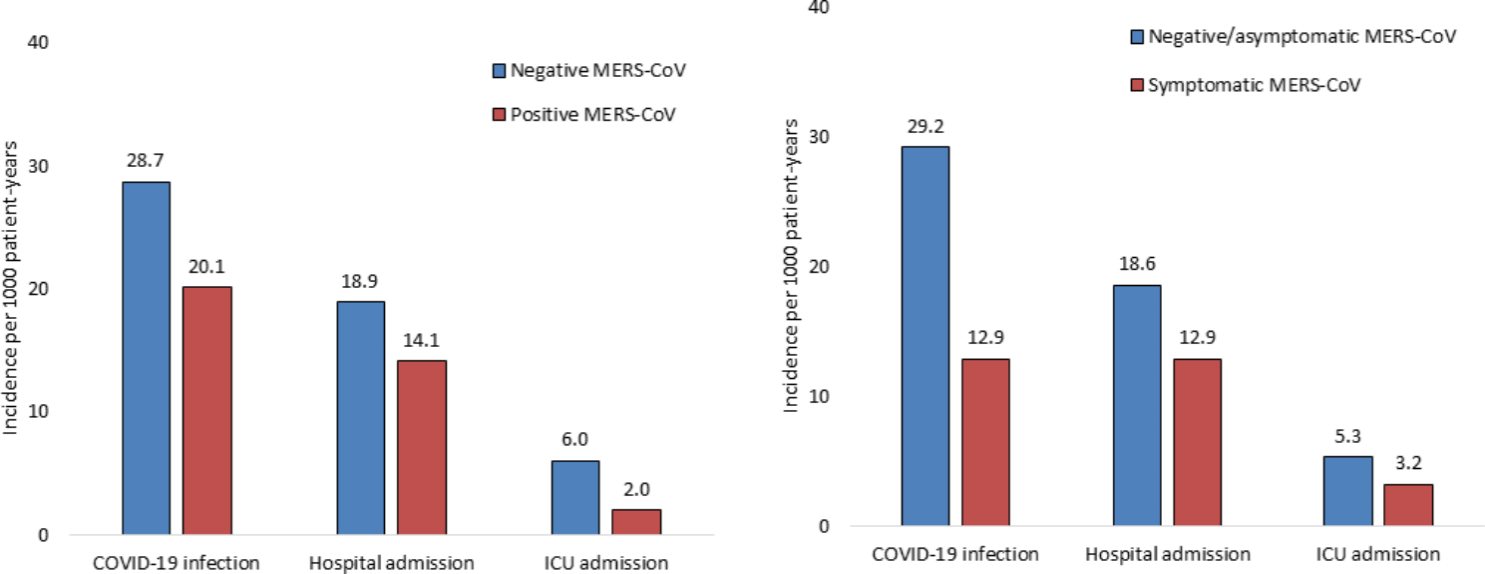
Incidence of the study outcomes per 1000 patient years at the end of follow up period by baseline MERS-CoV status

By the end of follow-up, the incidences per 1000 person-years of COVID-19 infection (20.1 versus 28.7), hospital admission (14.1 versus 18.9), and ICU admission (2.0 versus 6.0) were lower in patients with positive MERS-CoV compared with those with negative MERS-CoV, but without reaching statistical significance (Fig. 2). The risk of COVID-19 disease was reduced but still insignificant when comparing patients with symptomatic MERS-CoV versus those with negative or asymptomatic MERS-CoV. Table 3 shows detailed outcomes, including COVID-19 infection, disease severity, complications, mortality, hospital/ICU admission, use and duration of Mechanical Ventilation, and hospital/ICU length of stay. All outcomes were better in patients with positive or symptomatic MERS-CoV infection but without reaching statistical significance. For example, COVID-19 infection (6.6% versus 13.1%, p = 0.150), severe disease (1.6% versus 3.0%, p = 0.584), and mortality (1.6% versus 3.3%, p = 0.701) were lower in patients with symptomatic MERS-CoV versus those with negative or asymptomatic MERS-CoV. On the other hand, the follow-up time in patients with symptomatic MERS-CoV was significantly longer (5.5 versus 4.9 years, p = 0.002). There was no association between COVID-19 infection by followed up time, irrespective of MERS-CoV status.

**Table 3:** Study outcomes at the end of follow up period by baseline MERS-CoV status

	Negative MERS-CoV (N = 304)	Positive MERS-CoV (N = 93)	p-value	Negative/ asymptomatic MERS-CoV (N = 336)	Symptomatic MERS-CoV (N = 61)	p-value
**COVID-19 status**						
Negative	266 (87.5%)	83 (89.2%)	0.651	292 (86.9%)	57 (93.4%)	0.150
Positive	38 (12.5%)	10 (10.8%)		44 (13.1%)	4 (6.6%)	
**COVID-19 positivity by follow up duration**						
< 3 years	10 (14.7%)	2 (28.6%)	0.310	10 (14.7%)	2 (28.6%)	0.310
3–5 years	20 (17.4%)	3 (16.7%)	> 0.99	22 (18.5%)	1 (7.1%)	0.462
> 5 years	8 (6.6%)	5 (7.4%)	> 0.99	12 (8.1%)	1 (2.5%)	0.307
**COVID-19 severity of disease**						
Asymptomatic	238 (78.3%)	76 (81.7%)	0.595	262 (78.0%)	52 (85.2%)	0.584
Mild/moderate	56 (18.4%)	16 (17.2%)		64 (19.0%)	8 (13.1%)	
Severe/ARDS	10 (3.3%)	1 (1.1%)		10 (3.0%)	1 (1.6%)	
**Outcomes at the end of follow up**						
Hospital admission	25 (8.2%)	7 (7.5%)	0.829	28 (8.3%)	4 (6.6%)	0.801
ICU admission	8 (2.6%)	1 (1.1%)	0.692	8 (2.4%)	1 (1.6%)	> 0.99
ICU ventilation	4 (1.3%)	1 (1.1%)	> 0.99	4 (1.2%)	1 (1.6%)	0.568
Complications	10 (3.3%)	2 (2.2%)	0.740	10 (3.0%)	2 (3.3%)	> 0.99
Mortality	11 (3.6%)	1 (1.1%)	0.309	11 (3.3%)	1 (1.6%)	0.701
**Durations at the end of follow up** (median & IQR)						
Hospital length of stay (days)	8 (2–25)	3 (2–11)	0.211	6 (2–23)	8 (4–16)	0.784
ICU length of stay (days)	16 (14–17)	5 (5–5)	0.237	16 (14–17)	5 (5–5)	0.237
ICU ventilator days	14 (8–17)	5 (5–5)	0.157	14 (8–17)	5 (5–5)	0.157
Follow up years	4.7 (3.2–5.7)	5.6 (5.0–6.0)	< 0.001	4.8 (3.3–5.8)	5.5 (4.7-6.0)	0.002

### Adjusted outcomes

**Fig. 3:**
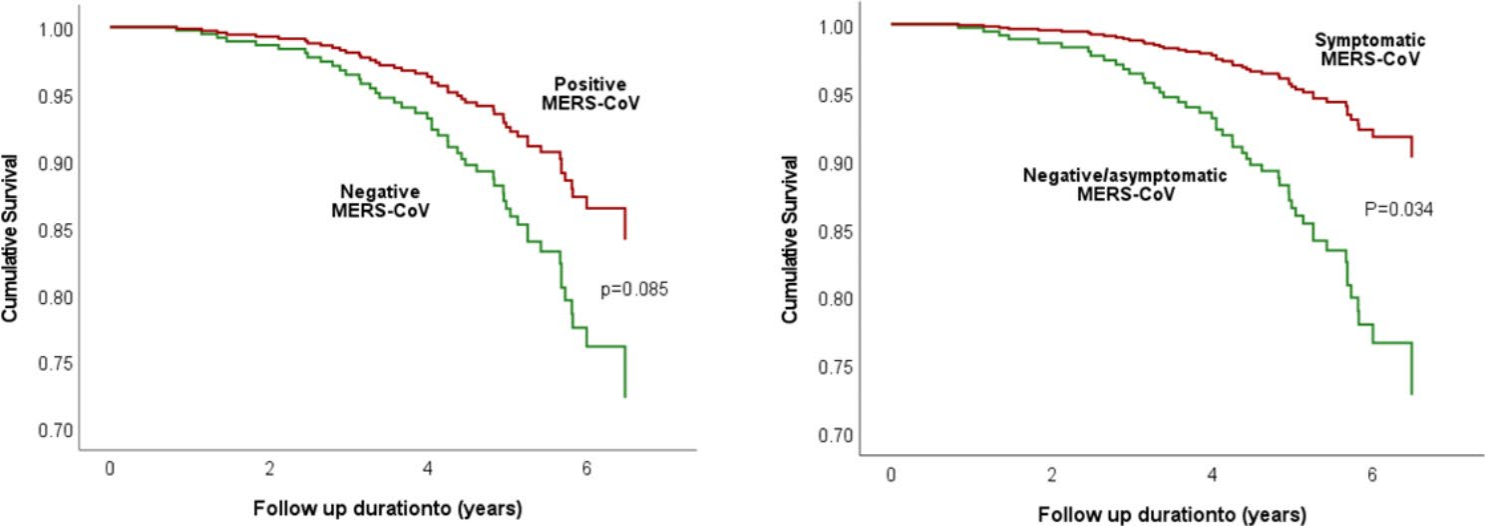
Adjusted Cox regression analysis of COVID-19 free survival at the end of follow up period by baseline MERS-CoV status*Adjusted for age at COVID, gender, and major comorbidity (hypertension, diabetes, heart diseases, lung diseases, and renal disease/hemodialysis)

As shown in Table 4, Cox regression analysis adjusted for the follow-up time in addition to age, gender, and major comorbidity at COVID assessment showed a marginally significant lower risk of COVID-19 infection (hazard ratio = 0.533, p = 0.085) and hospital admission (hazard ratio = 0.411, p = 0.061) in patients with positive MERS-CoV. Interestingly, the risk of COVID-19 disease was further reduced and became significant in patients with symptomatic MERS-CoV, COVID-19 infection (hazard ratio = 0.324, p = 0.034), and hospital admission (hazard ratio = 0.317, p = 0.042). Similarly, COVID-19-free survival at the end of the follow-up period was better in patients with positive MERS-CoV (p = 0.085) and those with symptomatic MERS-CoV (p = 0.034) (Fig. 3).

**Table 4:** Crude (unadjusted) and adjusted Cox regression models predicting the study outcomes at the end of follow up period by baseline MERS-CoV status

	Hazard ratio (HR)	95% lower confidence	95% lower confidence	p-value
**Comparing positive vs. negative MERS-CoV**				
**Crude models**				
COVID-19 infection	0.576	0.286	1.159	0.122
Hospital admission at the end of follow up	0.701	0.302	1.627	0.409
ICU admission at the end of follow up	0.356	0.044	2.855	0.331
Adjusted models*				
COVID-19 infection	0.533	0.261	1.09	0.085
Hospital admission at the end of follow up	0.411	0.162	1.041	0.061
ICU admission at the end of follow up	0.113	0.011	1.13	0.063
**Comparing symptomatic vs. negative or asymptomatic MERS-CoV**				
**Crude models**				
COVID-19 infection	0.391	0.14	1.088	0.072
Hospital admission at the end of follow up	0.669	0.234	1.911	0.453
ICU admission at the end of follow up	0.635	0.079	5.086	0.669
Adjusted models*				
COVID-19 infection	0.324	0.115	0.917	0.034
Hospital admission at the end of follow up	0.317	0.105	0.958	0.042
ICU admission at the end of follow up	0.369	0.039	3.534	0.387

*Adjusted for age at COVID, gender, and major comorbidity (hypertension, diabetes, heart diseases, lung diseases, and renal disease/hemodialysis)

## Discussion

The current study examined the risk of COVID-19 infection and its related outcomes among patients who survived MERS-CoV infection before the COVID-19 pandemic. There was a generally lower risk of COVID-19 disease and related hospitalization in patients with symptomatic MERS-CoV rather than just positive MERS-CoV, irrespective of symptoms. However, the risk only reached statistical significance in models adjusted for the follow-up time, age, gender, and significant comorbidity. Comparing the current finding is challenging due to scarce data testing MERS-CoV and COVID-19 cross-protection. We were able to identify only one similar study [7]. A retrospective cohort study in Saudi Arabia in 2020 showed a lower incidence of COVID-19 infection in patients with positive compared with negative MERS-CoV (24% versus 31%, p = 0.014) [7]. However, patients with positive MERS-CoV in that study had higher hospitalization and case fatality [7]. The later contradictory crude finding was probably caused by the fact that the positive MERS-CoV group in that study was significantly older and had a higher morbidity profile than the negative MERS-CoV group. This was further proven by eliminating the significance of case fatality by adjusting for age. Additionally, the authors did present a complete adjusted analysis for different study outcomes.

Consistent with current findings, a serological study in Sierra Leone showed significantly lower MERS-CoV antibodies among patients with confirmed COVID-19 infection compared with survivors of Lassa fever and Ebola and their contacts who did not have COVID-19 disease [20]. Additionally, some studies suggested the presence of cross-protection against COVID-19 infection in patients with previous seasonal coronaviruses [21]. A retrospective analysis of an extensive insurance database showed that adults with a possible coronaviruses-caused common cold as manifested by acute sinusitis, bronchitis, or pharyngitis during the last year had a lower risk of confirmed COVID-19 infection [21]. A significant limitation of that study was the lack of serological confirmation of previous coronavirus infection, which usually accounts for only 10–30% of common cold-related diagnoses.

The current finding confirms that symptomatic MERS-CoV infection protects more against COVID-19 than asymptomatic or negative MERS-CoV. This may be explained by the fact that severe MERS-CoV is associated with humoral and cellular immune responses that persist longer than subclinical or mild disease [15, 16]. Additionally, severe MERS-CoV disease is associated with a more robust immune response, including specific antibodies and memory CD4 T cells [15, 22, 23]. The more robust immune response in patients with severe MERS-CoV disease has been observed 6 to 24 months after infection in both Saudi Arabia [15] and South Korea [22]. On the other hand, the majority of patient with mild disease had undetectable antibody titre [22]. Unlike COVID-19, neutralization antibodies in patients with MERS-CoV are detectable up to 6 years after diagnosis [16]. Finally, cellular immunity including T cells, is essential for long-term immunity in both MERS-CoV AND COVID-19 [17, 18]. These immunologic findings may point to the possible cross-immunity against COVID-19 infection and severe outcomes among MERS-CoV survivals. Nevertheless, the lack of significant differences in ICU admission and mortality between the study groups of the current study may be related to their very low incidence (2.3% and 2.8%, respectively) compared with infection (12.1%).

The current study is considered the first to estimate the impact of previous MERS-CoV infection on the risk of COVID-19 disease and its related outcomes in a cohort design using adjusted analysis. Baseline cohorts were group-matched on age, gender, and year of testing. Both MERS-CoV and COVID-19 infection statuses were confirmed using PCR testing. The healthcare system where the data were collected has a unique experience with MERS-CoV [24, 25]. Nevertheless, the retrospective design may have introduced bias to the collected data. The single-center experience may limit the generalizability of the study findings, and the lack of serologic data may undermine the underlying mechanism of suggested protection. Healthcare workers who represented about 40% of the sample may have more screening opportunities than patients. The impact of over-screening is probably insignificant because it was similarly observed in MERS-CoV outbreak and COVID-19 pandemic. Additionally, the further analysis of the data by symptomatic status should eliminate most of such difference, as negative and asymptomatic patients were grouped as one group. The current data is considered unique and considerably contributes to our understanding regarding the cross-protection between coronaviruses.

In conclusion, patients with symptomatic MERS-CoV have a lower risk of COVID-19 infection and related hospitalization, especially after adjusting for demographic and comorbidity profiles. The current findings may indicate a partial cross-immunity between MERS-CoV and COVID-19 infection. These findings probably justify a national multicentre study using immunity markers to confirm cross-immunity and elaborate more on its mechanisms.

## Data Availability

The data that support the findings of this study are available from King Abdul Aziz Medical city. Restrictions apply to the availability of these data, which were used under license for this study. Data are available from the corresponding authors with the permission of King Abdul Aziz Medical city and Institutional Review Board of King Abdullah International Medical Research Center.

## Abbreviations

MERS-CoV: Middle East Respiratory Syndrome Coronavirus
KAMC-R: King Abdulaziz Medical City in Riyadh
MOH: Ministry of Health
HESN: Health Electronic Surveillance Network
